# Odorant Receptor *OR45a* Mediates Female-Specific Attraction to *cis*-Linalool Oxide in *Bactrocera dorsalis*

**DOI:** 10.3390/insects16111139

**Published:** 2025-11-07

**Authors:** Bibi Liang, Xianli Lu, Lu Xiao, Wang Miao, Shuchang Wang, Fengqin Cao, Jian Wen

**Affiliations:** 1 School of Tropical Agriculture and Forestry, Hainan University, Haikou 570228, China; 17361648743@163.com (B.L.); 18869855967@163.com (X.L.);; 2 Environment and Plant Protection Institute, Chinese Academy of Tropical Agricultural Sciences, Haikou 571101, China

**Keywords:** Tephritidae, attractants, olfactory receptors, ligand binding

## Abstract

*Bactrocera dorsalis* Hendel is a devastating invasive pest that costs billions of dollars in agricultural losses worldwide. Current control strategies rely heavily on male-specific attractants such as methyl eugenol, which are less effective against females, underscoring the need for female-targeted control approaches. Here, we investigated the molecular mechanisms underlying female attraction to cis-linalool oxide by functionally characterizing the odorant receptor *OR45a*, identifying it as a molecular target for female-oriented pest management. We conducted spatiotemporal expression analysis of *OR45a* in response to cis-linalool oxide, followed by RNAi and behavioral assays. Phylogenetic analysis of OR45a orthologs from 10 Dipteran species, combined with structural topology prediction and solvent-accessible surface area (ASA) analysis, helped identify functional domains and residues. Site-directed mutagenesis and two-electrode voltage clamp (TEVC) recordings validated receptor–ligand interactions. Results showed that *OR45a* was specifically upregulated in antennae, with peak expression at 10 days post-eclosion, coinciding with oviposition periods. RNAi significantly reduced *OR45a* transcript levels and female behavioral responses to cis-linalool oxide. Phylogenetic analysis showed that OR45a is highly conserved within Tephritidae but diverges from Drosophilidae, with closest similarity to *Anastrepha ludens*, indicating ecological specialization. Structural modeling predicted a canonical seven-transmembrane architecture with three extracellular loops forming the ligand-binding pocket. Among five key residues identified, Leu122 and Ile146 were essential for ligand recognition, while Tyr107 contributed to protein stability. These findings reveal a female-specific odorant receptor mechanism in *B. dorsalis* and provide molecular targets for *OR45a*-based attractants, addressing a critical gap in female-focused pest management.

## Simple Summary

The oriental fruit fly (*Bactrocera dorsalis*) is a major agricultural pest that damages fruits around the world. Most current control methods use chemicals that attract only males, leaving females—who lay the eggs that cause infestations—largely unaffected. In this study, we discovered how female *B. dorsalis* detect a natural scent compound called *cis*-linalool oxide. We identified a specific odorant receptor, called OR45a, that allows females to sense this compound. By reducing the activity of this receptor, we found that females became less attracted to *cis*-linalool oxide. Our molecular and structural analyses revealed key parts of OR45a that determine how it recognizes this scent. These results improve our understanding of female *B. dorsalis* smell perception and open new possibilities for developing lures or control strategies specifically targeting female flies.

## 1. Introduction

The oriental fruit fly, *Bactrocera dorsalis* Hendel, is a major invasive pest threatening fruit and vegetable production in tropical and subtropical regions [1,2,3,4]. Its high dispersal and reproductive capacity have caused substantial economic losses worldwide, estimated at tens of billions of US dollars, including ~US$1 billion annually in Southeast Asia and potential impacts exceeding CN¥20 billion in South China [5,6,7]. Climate change and international trade exacerbate its spread into higher latitudes, emphasizing the urgent need for effective control strategies [8]. Conventional chemical controls face limitations due to pesticide resistance and non-target organism mortality [9,10]. Methyl eugenol (ME), a male-attractant widely used for monitoring and control, exhibits low trapping efficiency for females (~9%) and thus cannot fully disrupt reproduction [11], highlighting the importance of developing female-specific attractants.

Recent studies have advanced understanding of male olfactory mechanisms. CRISPR/Cas9-mediated Orco mutants and DREAM technology identified BdorOR94b1 as an ME-specific receptor [12]. And BdorOBP2 plays an indispensable role in the perception of methyl eugenol by mature males of *B. dorsalis* [13]. Transcriptomic analyses revealed that *OBP49a* and *OBP83b* synergistically mediate pheromone recognition through key residues His^56^ and Lys^72^, while *CSP3* is antenna-specifically upregulated after odor stimulation and binds ME and *β*-caryophyllene with high affinity [14,15]. In contrast, female olfactory mechanisms remain poorly understood. Evidence suggests *BdorOBP83a* participates in *α*-pinene recognition, and gut symbiont-derived phenylethanol activates female receptor BdorOR71a to regulate oviposition [16,17].

Insect odorant receptors (ORs) are specialized proteins that enable insects to detect and discriminate a wide range of chemical cues in their environment, playing a crucial role in behaviors such as finding food, mates, and oviposition sites [18]. These receptors typically function as heteromeric complexes, consisting of a variable odorant-sensing subunit and a highly conserved co-receptor called Orco. The OR–Orco complex forms a ligand-gated ion channel, where the odorant-sensing subunit binds specific odorants, and Orco acts as a scaffold and ion channel component, facilitating signal transduction upon odorant binding [19,20]. Structural studies have revealed that ligand binding induces conformational changes in the complex, leading to channel opening and ion influx, which is essential for olfactory signaling [20]. In *B. dorsalis*, *ORs* exhibit sex- and context-specific functions: *OR94b1* mediates male ME attraction, whereas *OR13a*, *OR82a*, *OR88a*, and benzothiazole-sensitive receptors (*OR43a-1*, *OR63a-2*) contribute to oviposition-related odor detection [15,21,22].

Linalool oxide, an epoxidized monoterpene from *Camellia sinensis* and *Lavandula angustifolia*, exhibits complex floral–fruity–woody aromas [23] and functions as a female-specific attractant for *B. dorsalis* [24]. It also attracts parasitoid wasps in tritrophic interactions and displays sexually dimorphic effects, with females showing stronger antennal responses than males [25,26]. Despite these findings, the molecular mechanisms underlying female-specific perception remain largely unknown.

Here, we investigate the role of *OR45a* in mediating *cis*-linalool oxide detection in female *B. dorsalis*. Our focus on *OR45a* stemmed from preliminary observations that *cis*-linalool oxide is a key component of the honeydew produced by mealybug, *Planococcus lilacinus*, which elicits strong attraction in female but not male *B. dorsalis* [24]. Moreover, our previous transcriptomic analyses revealed that *OR45a* expression was significantly upregulated in female antennae but not in males (unpublished data). These results suggested that OR45a may encode an olfactory receptor tuned to cis-linalool oxide, thereby mediating female-specific chemotaxis toward this compound. Therefore, in this study we: (1) characterize OR45a’s spatiotemporal expression across developmental stages and tissues; (2) assess its necessity for attraction via RNA interference and wind-tunnel assays; (3) examine its evolutionary conservation and predict key functional residues; and (4) validate residue-specific ligand recognition using site-directed mutagenesis and heterologous expression. This study addresses a critical gap in female olfactory biology and provides molecular targets for OR45a-based female attractants, offering a strategy to overcome limitations of male-focused traps and improve pest management.

## 2. Materials and Methods

### 2.1. Insect Rearing

Larvae of *B. dorsalis* were collected from a mango orchard at the Haidian campus of Hainan University, Haikou, China. Laboratory colonies were established and maintained for at least 30 generations under controlled conditions to ensure genetic stability. Larvae were reared on an artificial diet composed of yeast extract (5%), sugar (10%), banana pulp (20%), filter paper (2%), concentrated hydrochloric acid (0.1%), sodium benzoate (0.2%), cornstarch (15%), and water (47.7%). Adults were provided with a diet containing yeast extract (3%), sugar (10%), agar (1%), honey (5%), and water (81%). All insects were maintained at 28 ± 1 °C, 70 ± 5% relative humidity, and a 16L:8D photoperiod in climate-controlled chambers.

### 2.2. Spatiotemporal Expression Analysis of OR45a

To investigate the spatiotemporal expression of OR45a following exposure to cis-linalool oxide, thirty newly emerged (within 24 h after eclosion) female flies were placed in mesh cages (25 × 25 × 25 cm) and exposed to cis-linalool oxide for 1, 5, 10, or 15 days. A total of 5 mL of 10% cis-linalool oxide (dissolved in dimethyl sulfoxide, DMSO) was applied to a 10 cm glass Petri dish. The dish, positioned centrally at the bottom of the cage, was sealed with Parafilm M and perforated with thirty evenly spaced 1–2 mm holes to allow controlled volatile release. Control groups received the same volume of DMSO.

After exposure, flies were anesthetized on ice for 5 min and dissected in cold PBS (4 °C) under a stereomicroscope. Three tissue types were collected: antennae (100 pairs per biological replicate), legs (all six legs from 50 individuals per replicate), and ovipositors (50 per replicate). Each treatment × tissue × time point combination included three biological replicates. Samples were immediately flash-frozen in liquid nitrogen and stored at -80 °C until RNA extraction.

Total RNA was extracted using TRIzol reagent (Invitrogen, Carlsbad, CA, USA) following the manufacturer’s protocol, combined with on-column DNase I treatment (Qiagen, Hilden, Germany) to remove genomic DNA contamination. RNA concentration and purity were assessed using a NanoDrop 2000 spectrophotometer (Thermo Fisher Scientific, Waltham, MA, USA), and integrity was evaluated with an Agilent 2100 Bioanalyzer (Agilent Technologies, Santa Clara, CA, USA) using RNA 6000 Nano kits. Only samples with A260/A280 ratios between 1.8 and 2.0 and RNA integrity numbers ≥ 7.0 were used for downstream analysis.

First-strand cDNA was synthesized from 1 μg of total RNA using the PrimeScript™ RT Reagent Kit with gDNA Eraser (Takara, Kyoto, Japan), according to the manufacturer’s instructions. No reverse transcriptase controls (–RT) were included to confirm the absence of genomic DNA contamination.

Quantitative PCR was performed in 20 μL reactions containing SYBR Green Master Mix (Takara, Japan), gene-specific primers (final concentration 0.3 μM), and diluted cDNA template (equivalent to 10 ng RNA input). Thermal cycling conditions were: initial denaturation at 95 °C for 30 s, followed by 40 cycles of 95 °C for 5 s and 60 °C for 30 s. Primer (forward: TATTGAATACCGGCGTGCTC; reverse: ATAGATGGCAACCGCTGTCC) specificity was confirmed by melting curve analysis and sequencing of PCR products. Primer efficiency was determined using a five-point serial dilution of cDNA and ranged between 90% and 110%. GAPDH was selected as the reference gene after validation of its expression stability across tissues and treatments using geNorm analysis.

Relative expression levels were calculated using the 2^–ΔΔCt^ method with error propagation. Data normality and homogeneity of variances were assessed using the Shapiro–Wilk and Levene’s tests, respectively. All qPCR-derived relative expression data of *OR45a* were grouped by tissue type (antennae, legs, ovipositor) and time point (1, 5, 10, 15 days). A simple t-test was performed to compare the differences between treatment and control groups, and results were considered significant at *p* < 0.05.

### 2.3. RNA Interference and Behavioral Bioassay

dsRNA design and synthesis: Gene-specific primers (sense strand sequence: GUUACAUUGUGGUGCUCAAGG; antisense strand sequence: UUGAGCACCACAAUGUAACUG), which target a 400 bp fragment within the open reading frame of *OR45a*, were designed using the DSIR online tool (http://biodev.extra.cea.fr/DSIR/DSIR.html, accessed on 1 August 2025). Potential off-target effects were excluded by BLAST (https://blast.ncbi.nlm.nih.gov/Blast.cgi, accessed on 1 August 2025) analysis against the *B. dorsalis* genome (E-value < 1 × 10^–5^). PCR amplification was performed using recombinant plasmids containing OR45a sequences cloned into pGEM-T vectors as templates. PCR products were verified by agarose gel electrophoresis and Sanger sequencing. Double-stranded RNA (dsRNA) synthesis was conducted using the RiboMAX^™^ Express T7 In Vitro Transcription System (Promega, Madison, WI, USA) in 20 μL reactions containing 10 μL RiboMAX^™^ Express T7 2× Buffer, 1 μg linearized DNA template, 2 μL T7 Enzyme Mix, and nuclease-free water. Reactions were incubated at 37 °C for 4 h, then heated to 70 °C for 10 min and gradually cooled over 20 min to facilitate annealing. To remove residual DNA and single-stranded RNA, 1 μL RNase Solution and 1 μL RQ1 RNase-Free DNase (Promega) were added, followed by incubation at 37 °C for 1 h. dsRNA was precipitated by adding 2 μL 3 M sodium acetate (pH 5.2) and 20 μL isopropanol, incubated on ice for 5 min, and centrifuged at 16,000× *g* for 10 min at 4 °C. Pellets were washed twice with 500 μL 70% ethanol, air-dried for 10 min, and dissolved in 20 μL nuclease-free water. Concentration and purity were measured with a NanoDrop 2000 (Thermo Fisher Scientific), and integrity confirmed by 1% agarose gel electrophoresis. Absence of RNase contamination was verified by incubating dsRNA at 37 °C for 1 h followed by gel analysis.

Microinjection: Ten-day-old female *B. dorsalis* were selected based on preliminary data showing highest *OR45a* expression at this stage. Flies were anesthetized with CO_2_ and injected with 1 μL *dsRNA* solution (5 μg/μL) between the seventh and eighth abdominal segments using a Nanoject III programmable nanoliter injector (Drummond Scientific, Broomall, PA, USA) equipped with a pulled glass capillary needle (tip diameter ~10 μm). Injection speed was set to 50 nL/s to minimize tissue damage. Post-injection, flies were allowed to recover under standard rearing conditions with ad libitum access to food and water. Mortality was monitored for 48 h. Three control groups were included: *dsGFP* injection (negative control for RNAi specificity), water injection (vehicle control), and non-injected flies (handling control). Knockdown efficiency assessment: At 48 h post-injection, five whole female flies per replicate (six biological replicates per treatment) were collected for RNA extraction using TRIzol reagent with on-column DNase treatment. cDNA synthesis and qPCR were performed as described previously. Primers targeting *OR45a* and the reference gene GAPDH were used. Knockdown efficiency was calculated relative to *dsGFP*, water, and non-injected controls, with >70% transcript reduction considered successful.

Behavioral bioassay: Behavioral responses to cis-linalool oxide were evaluated using a custom-built wind tunnel (150 × 40 × 50 cm; Figure S1) adapted from Wen et al. [24]. The tunnel was divided into three zones: the odor source zone (20 cm, upwind), the central flight area (110 cm), and the release zone (20 cm, downwind). Laminar airflow was maintained at 0.3 m/s, the temperature at 27–28 °C, and relative humidity at 60–70%. Airflow uniformity was confirmed by anemometer measurements at multiple points. The odor source was the same as described above and consisted of a Petri dish (diameter = 10 cm) containing 5 mL of 10% cis-linalool oxide in DMSO. The dish was sealed with Parafilm M and perforated with 1–2 mm holes to ensure uniform volatile release. The entire odor source was placed centrally at the bottom of the odor source zone. Control trials used DMSO alone. At 48 h after dsRNA injection, 30 females from each treatment group (dsOR45a, dsGFP, water-injected, and non-injected) were introduced into the release zone and acclimated for 2 min. Flies entering the odor source zone within 10 min were recorded as positive responders. Observations were conducted under red light (>650 nm) to minimize visual disturbance. The positions of odor and control sources were alternated between trials to avoid positional bias. The apparatus was cleaned with 70% ethanol and air-dried between trials. Observers were blinded to treatment identities. Each treatment group was tested in 10 independent biological replicates.

Statistical analysis: Gene expression, mortality rates and behavioral responses were analyzed using the Kruskal–Wallis test due to non-normality (assessed by Shapiro–Wilk test, *p* < 0.05) across treatments: Control, water, *dsGFP*, and *dsOR45a*, followed by Dunn’s multiple comparisons with Bonferroni correction.

### 2.4. Evolutionary Analysis and Sequence Conservation of OR45a

This study systematically investigated the sequence conservation and phylogenetic relationships of insect OR45a proteins across multiple species. A detailed examination of its phylogeny and sequence divergence helps reveal conserved functional regions and adaptive variations in an evolutionary context.

The entire analysis pipeline was implemented in R. We collected OR45a protein sequences from *B. dorsalis* and other representative insect species from NCBI (Table S1). The sequences were imported in FASTA format. We loaded the required R packages (R version 4.5.1) for sequence processing (“Biostrings”, “msa”, “seqinr”), multiple sequence alignment (“msa”), phylogenetic analysis (“phangorn”, “ape”), and data visualization (“ggplot2”, “ggtree”, “viridis”).

Multiple sequence alignment was performed using the “msa” package with the ClustalW algorithm to generate full-length alignments of all input sequences. The resulting alignment was converted into an amino acid character matrix, which served as the basis for subsequent analyses. Based on this matrix, we quantitatively assessed the conservation at each alignment position by calculating the frequency of the most common amino acid residue. This conservation score reflects the degree of sequence conservation across all species at each site.

$$C_{i}=\frac{\max(\mathrm{count}(r\in {\mathrm{col}}_{i}))}{n_{i}}$$

Here, C_i_ denotes the conservation score for the i-th position, calculated as the count of the most frequent amino acid residue (numerator) divided by the total number of valid residues at that position (n_i_). The score ranges from 0 to 1, with higher values indicating greater conservation across all input species.

In the phylogenetic analysis, the aligned protein sequences were first converted into the phyDat format using the “phangorn” package in R (R version 4.5.1). We then computed a pairwise evolutionary distance matrix under the JTT (Jones–Taylor–Thornton) substitution model using the maximum likelihood approach implemented in the dist.ml () function. The JTT model is a widely used empirical amino acid substitution model derived from observed protein evolution data. Using the resulting distance matrix, we constructed an initial unrooted phylogenetic tree with the Neighbor-Joining (NJ) algorithm, which infers evolutionary relationships based on pairwise distances.

To evaluate the robustness of the inferred tree topology, we performed bootstrap analysis with 100 replicates. For each bootstrap replicate, the aligned sequences were resampled with replacement, evolutionary distances recalculated, and NJ trees reconstructed. The bootstrap support value for each internal node was calculated as the proportion of replicates in which that node appeared. The bootstrap analysis was conducted using the bootstrap.phyDat () function from the “phangorn” package, which automates the resampling and tree reconstruction process.

$$\mathrm{Bootstrap}\;\mathrm{Support}(\%)=\frac{\mathrm{Clade}\;\mathrm{repeated}\;\mathrm{times}\;\mathrm{in}\;\mathrm{bootstrap}\;\mathrm{trees}\;}{100}\;\times \;100\%$$

### 2.5. Identification of Key Functional Residues

To identify conserved residues with potential structural or functional significance, we integrated sequence conservation data with transmembrane topology annotations, marked highly conserved amino acids located in functionally critical regions as preliminary candidates, and then performed solvent-accessible surface area (ASA) analysis to select key functional residues.

First, the secondary topology of the *B. dorsalis* OR45a protein was predicted using TopCons (https://topcons.cbr.su.se/, accessed on 20 September 2025), an integrated prediction platform that combines multiple algorithms (including OCTOPUS, Philius, PolyPhobius, etc.) to generate a consensus topology. It is commonly used for predicting GPCR and OR-type proteins, providing more robust results. These analyses identified transmembrane helices (TMs), along with intracellular loops (ILs) and extracellular loops (ELs) connecting them.

We combined this topology information with conservation scores derived from the multiple sequence alignment results obtained previously. Our focus was on extracellular loops and the termini of transmembrane segments, as these regions are most likely to form ligand-binding pockets located at TMH–EL junctions, which may be high-potential structural–functional regions. Each residue was classified based on whether it fell within these regions. Residues with a conservation score ≥0.9 located in these regions were designated as preliminary candidates. The threshold of 0.9 was chosen to ensure high confidence in conservation, consistent with previous studies.

To assess whether these candidates exhibit high surface accessibility, indicating potential ligand or solvent interaction, we performed solvent-accessible surface area (ASA) analysis based on the protein’s three-dimensional structure. The ASA score estimates a residue’s relative exposure in the spatial conformation and is widely used in structural biology to identify functional sites and antigenic epitopes.

Because standard ASA calculations require detailed solvent exposure measurements or spherical probe algorithms, we implemented a simplified method for rapid screening by loading the PDB structure file of BdorOR45a, predicted by AlphaFold2 (see Supporting Information Figures S2 and S3), using the “bio3d” package in R. For each target residue, we located its Cα atom coordinates, counted the number of neighboring Cα atoms within an 8 Å radius (local density), and recorded the shortest Euclidean distance to any other Cα atom (nearest neighbor distance). These two measures were combined into a simplified ASA score, where larger values indicate greater surface exposure:

$${ASA}_{i}\;=\;max(0,20-N_{i})\;+\;2\;\times \;D_{i}$$

Here, *N_i_* denote the number of neighboring Cα atoms within an 8 Å radius around the *i*-th residue. Similarly, *D_i_* represents the minimum Euclidean distance (in Å) from the *i*-th residue to its nearest neighboring Cα atom.

A constant term is included in the ASA scoring formula to control the scoring range and ensure a linear distribution of scores, which facilitates normalization for comparison across residues. After calculating the ASA scores for all residues, the values were linearly normalized to the range [0, 100], with higher scores indicating greater solvent accessibility. Residues with high ASA scores were chosen as key residues.

### 2.6. Site-Directed Mutagenesis and Functional Validation

Mutagenesis and molecular cloning: Five residues identified from the structural and conservation analyses (Thr103, Tyr107, Val114, Leu122 and Ile146) were selected for functional characterization. Site-directed point mutations were generated by PCR-based overlap extension (QuikChange-style) using mutagenic primer pairs that place the desired codon centrally within each primer. Mutagenic primers are listed in Table S2. PCRs were performed using Phusion High-Fidelity DNA Polymerase (New England Biolabs, Ipswich, MA, USA) in 50 µL reactions containing 1× Phusion HF buffer, 200 µM dNTPs, 0.5 µM of each primer, 10–50 ng plasmid template and 0.5–1 U Phusion polymerase. Thermal cycling conditions were: initial denaturation 98 °C for 30 s; 25 cycles of 98 °C for 10 s, annealing (gradient 55–68 °C) for 20–30 s, and extension at 72 °C with 30 s per kb of plasmid length; final extension at 72 °C for 5 min. After amplification, PCR products were treated with DpnI (New England Biolabs) at 37 °C for 1 h to digest parental methylated DNA, and 2–5 µL of the digestion was transformed into chemically competent *E. coli* (DH5α). Colonies were screened by colony PCR and confirmed by Sanger sequencing covering the entire OR45a coding region to verify the intended mutation and to exclude secondary mutations. Confirmed mutant and wild-type OR45a coding sequences were subcloned into the pT7TS expression vector using XhoI and NotI restriction sites downstream of the T7 promoter and upstream of the V5 epitope tag. Plasmids were validated by restriction mapping and full-length sequencing. Plasmids were linearized with SpeI and used as templates for in vitro transcription. Capped cRNAs were synthesized using the mMESSAGE mMACHINE T7 kit (Thermo Fisher Scientific) according to the manufacturer’s instructions. cRNA quantity and integrity were verified by NanoDrop spectrophotometry and agarose gel electrophoresis.

Protein expression validation: To confirm the expression of OR45a and its site-directed mutants, seven treatment groups were prepared: (1) Empty vector (negative control), (2) Wild-type (WT) OR45a, and five OR45a variant constructs (Thr103, Tyr107, Val114, Leu122, and Ile146). *Xenopus laevis* oocytes were injected with the respective capped cRNAs, and after 48 h post-injection, groups of oocytes were lysed in RIPA buffer supplemented with protease inhibitors (Roche, Basel, Switzerland). Total protein concentrations were determined using the BCA assay, and equal amounts of protein (20 µg per lane) were subjected to SDS–PAGE and transferred to PVDF membranes. Membranes were blocked in 5% nonfat milk/TBST and probed with mouse anti-V5 antibody (1:2000; Thermo Fisher Scientific) to detect V5-tagged OR45a, and with rabbit anti-β-actin antibody (1:5000) as a loading control. HRP-conjugated secondary antibodies were applied, and bands were visualized by enhanced chemiluminescence (ECL).

Oocyte preparation and microinjection: Stage V–VI *X. laevis* oocytes were defolliculated enzymatically with 2 mg/mL collagenase type II in Ca^2+^-free buffer (82.5 mM NaCl, 2 mM KCl, 1 mM MgCl_2_, 5 mM HEPES, pH = 7.5) for 1–2 h at room temperature with gentle agitation, then extensively washed with ND96 buffer to remove enzyme. Equal amounts of *OR45a* (wild-type or mutant) and *Orco* cRNAs (50 ng each in 50 nL) were co-injected per oocyte using a Nanoject II microinjector (Drummond Scientific Company, Broomall, PA, USA). Water-injected oocytes, and oocytes injected with *OR45a*+*Orco* alone were included as controls. Two days post-injection oocytes were used for two-electrode voltage clamp (TEVC) assays. Injected oocytes were incubated at 18 °C in ND96 buffer (96 mM NaCl, 2 mM KCl, 1.8 mM CaCl_2_, 1 mM MgC_2_, 5 mM HEPES, pH = 7.5) supplemented with 50 µg/mL gentamicin for 48 h before recording. Functional responses to *cis*-linalool oxide and DMSO (vehicle control) were recorded using a two-electrode voltage clamp (TEVC) system (Axon Instruments, pClamp 10.7 software, San Jose, CA, USA). Oocytes were clamped at −80 mV. Dose–response curves were generated by applying ligand concentrations of 1%, 10%, and 20%, with washout periods between applications. Maximum current amplitude (I_max_) and EC_50_ values were calculated. Ten oocytes per construct were tested to ensure reproducibility.

## 3. Results

### 3.1. Spatiotemporal Expression Profile of OR45a in Response to cis-Linalool Oxide

**Figure 1:**
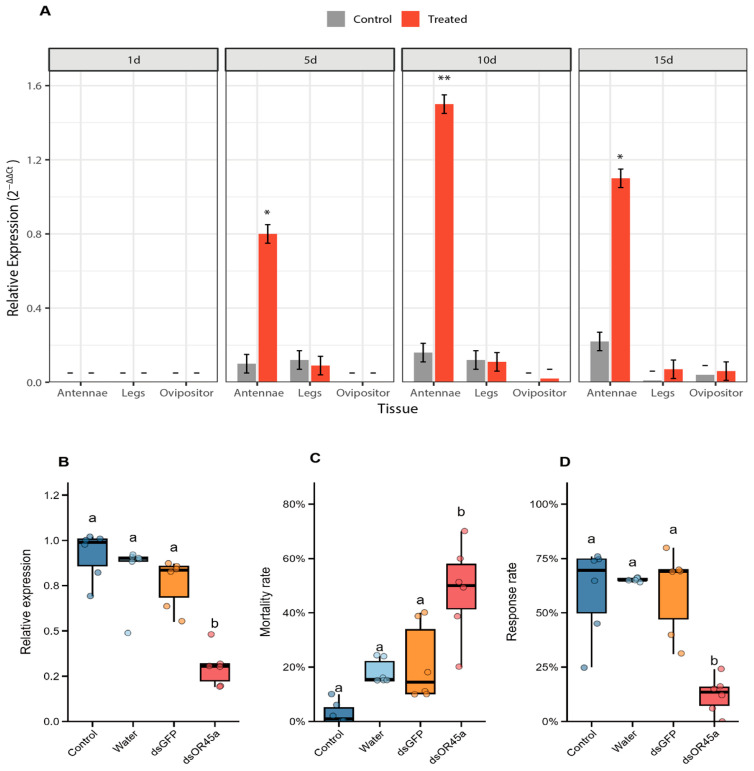
Relative expression of *OR45a* in the antennae, legs, and ovipositors of *B. dorsalis* exposed to *cis*-linalool oxide for 1, 5, 10, and 15 days post-eclosion (**A**). Asterisks indicate significant differences between control and cis-linalool oxide treatments (* *p* < 0.05; ** *p* < 0.001). Relative expression of *OR45a* after RNAi treatment in the 48 h after injection (**B**) and its effect on *B. dorsalis* mortality within 48 h after injection (**C**). Effect of RNAi on female response to *cis*-linalool oxide (**D**). Different lowercase letters indicate significant differences among treatments (Control, water, *dsGFP*, *dsOR45a*).

In the antennae, no significant difference was observed between the treatment and control groups after 1 day of exposure (t = 0.10, *p* = 0.923; Figure 1A). In contrast, highly significant differences were detected after 5 days (t = 11.26, *p* = 0.020), 10 days (t = 35.21, *p* < 0.001), and 15 days (t = 13.57, *p* = 0.012) of exposure. For other tissues and exposure durations, no significant differences in *OR45a* expression were observed compared with the control group without cis-linalool oxide treatment (all t ≤ 2.13, *p* ≥ 0.129).

### 3.2. Functional Validation of OR45a via RNA Interference

RNAi resulted in significant differences in *OR45a* transcript levels among the four groups (χ^2^ = 15.70, df = 3, *p* = 0.001; Figure 1B). Post hoc comparisons indicated that the *dsOR45a* group differed significantly from both the Control and Water groups, whereas no significant differences were found among Control vs. *dsGFP*, Water vs. *dsGFP*, or *dsGFP* vs. *dsOR45a*. Mortality rate also varied significantly among groups (χ^2^ = 17.40, df = 3, *p* < 0.001; Figure 1C), with a significant increase observed only in the *dsOR45a* group compared to other treatments. Injection with water and *dsGFP* also caused a significantly higher mortality rate compared to the control. Behavioral response rate showed a similar pattern (χ^2^ = 13.4, df = 3, *p* = 0.004; Figure 1D), with significant decreases in *dsOR45a* relative to Control and *dsGFP*, while other pairwise comparisons were nonsignificant (Figure 1D).

### 3.3. Sequence Alignment and Conservation Analysis of OR45a Orthologs

**Figure 2:**
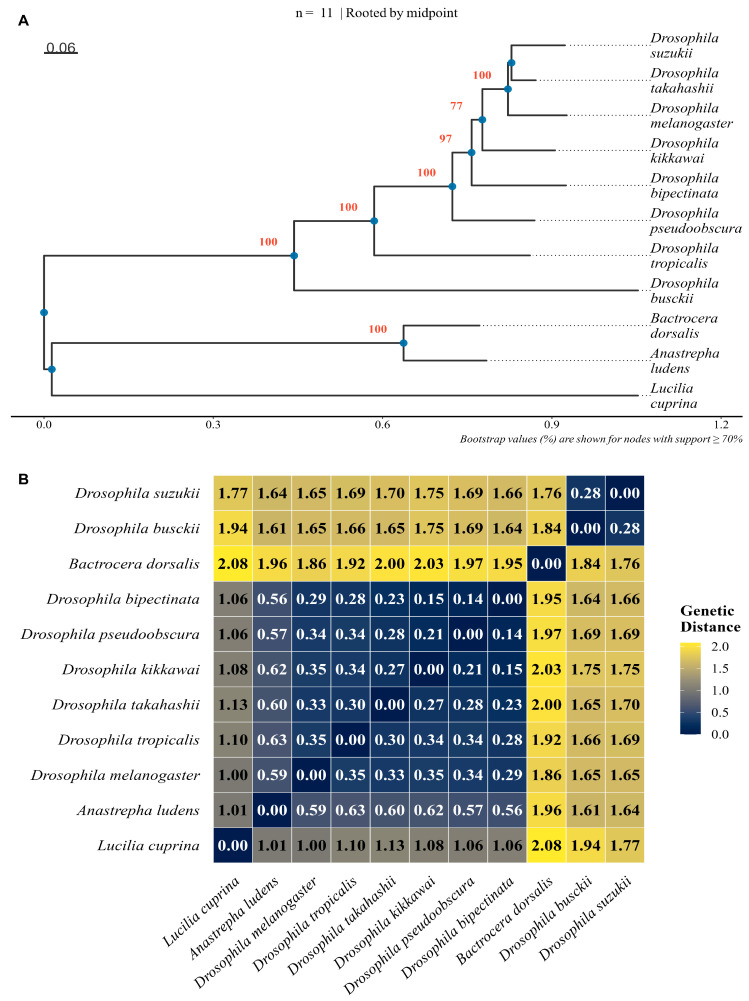
(**A**) Phylogenetic tree of *B. dorsalis* OR45a together with OR45a orthologs from ten other Dipteran species. (**B**) Heatmap of pairwise genetic distances among these Dipteran species.

The amino acid sequence of *B. dorsalis* OR45a and its orthologs from 10 Dipteran species were analyzed (Table S1). The dataset included representatives from Tephritidae (e.g., *Anastrepha ludens*) and Drosophilidae (e.g., *Drosophila melanogaster*, *D. suzukii*, *D. pseudoobscura*), with sequence lengths ranging from 315 to 415 amino acids (mean = 375.7). Multiple sequence alignment yielded 424 aligned positions. The phylogenetic tree of OR45a homologs shows that the OR45a of *B. dorsalis* clusters closely with that of *A*. *ludens*, forming a highly bootstrap value (100%). In contrast, the OR45a of species in the genus *Drosophila* are clearly differentiated. *Lucilia cuprina* serves as an outgroup and is clearly separated from the other species, thus validating the reliability of the phylogenetic tree (Figure 2A). The mean pairwise genetic distance estimated under the JTT model was 1.1268, with a maximum of 2.0825. Pairwise analysis also revealed that *B. dorsalis* OR45a was most similar to that of *D. suzukii* (distance = 1.76). By contrast, large genetic distances were observed between *B. dorsalis* and *Lucilia cuprina* (distance = 2.08; Figure 2B).

**Figure 3:**
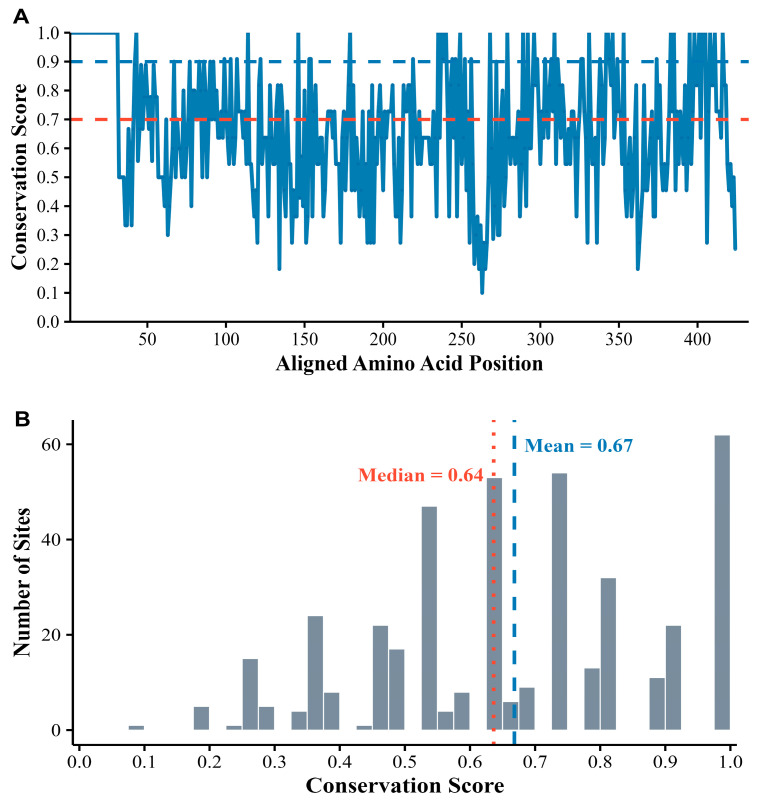
Conservation analysis of OR45a homologs from 11 species. (**A**) Conservation scores at each amino acid position. The red and blue dashed lines represent conservation thresholds of 0.7 and 0.9, respectively. (**B**) The conservation scores are distributed with a median and mean of 0.64 and 0.67, respectively.

Among these sites, 84 (19.8%) were highly conserved (>90% identity), whereas 230 (54.2%) were variable (≤70% identity; Figure 3A). The mean and median conservation scores were 0.67 and 0.64, respectively (Figure 3B).

### 3.4. Prediction and Analysis of the Transmembrane Topology of OR45a

**Figure 4:**
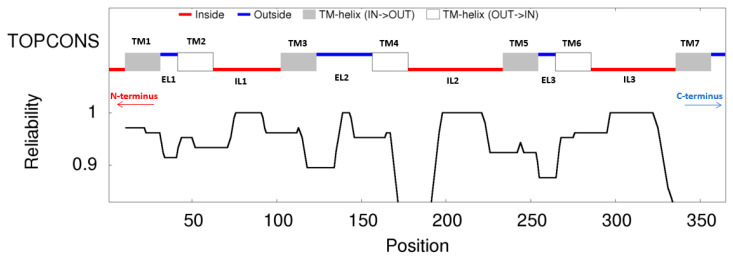
Consensus transmembrane topology model of *B. dorsalis* OR45a predicted by TOPCONS. TM1–TM7 represent transmembrane helices 1–7, EL1–EL3 represent extracellular loops 1–3, and IL1–IL3 represent intracellular loops 1–3.

TOPCONS predicted that OR45a is a 356-amino-acid membrane protein with seven transmembrane helices located at 11–31, 42–62, 103–123, 157–177, 234–254, 265–285, and 336–356 (Figure 4 and Figure S4). The N-terminus is cytoplasmic and the C-terminus extracellular, consistent with the inverted topology of insect odorant receptors. No cleavable N-terminal signal peptide was predicted; The helices define three extracellular loops (EL1: 32–41, EL2: 124–156, EL3: 255–264) and three intracellular loops (IL1: 63–102, IL2: 178–233, IL3: 286–335), followed by a short extracellular C-tail. These extracellular loops, together with the extracellular ends of TM3–TM7, are plausible contributors to ligand recognition and binding.

### 3.5. Conservation and Solvent-Accessible Surface Area Analysis of Key Residues

**Figure 5:**
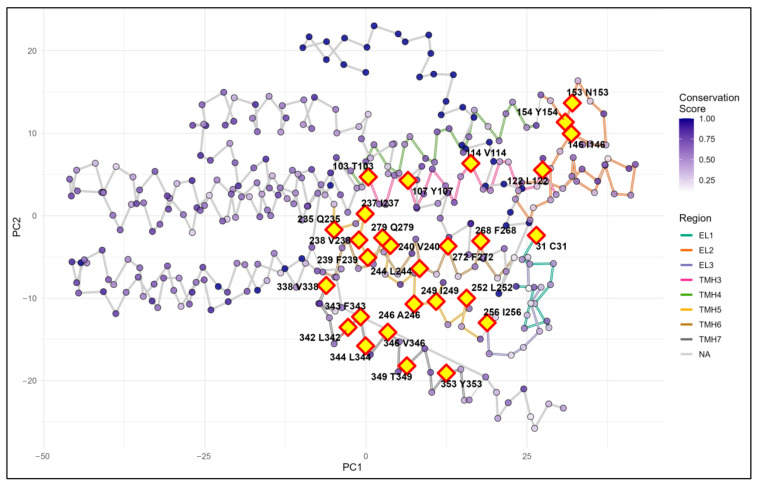
Predicted two-dimensional structure of *B. dorsalis* OR45a protein with key functional regions and candidate residues (red diamond) highlighted. PC1 and PC2 represent the first two principal components derived from PCA of Cα atomic coordinates obtained from the PDB, providing a two-dimensional visualization of protein structure. TMH3–TM7 represent transmembrane helices 3–7, EL1–EL3 represent extracellular loops 1–3, and NA represents the non-binding region.

**Figure 6:**
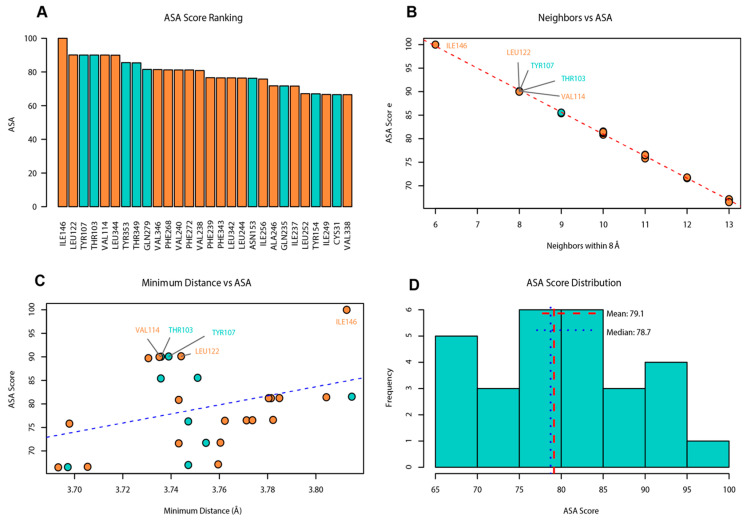
(**A**) ASA score ranking of 28 candidate residues. (**B**) Neighboring atoms of each residue within 8 Å. (**C**) Nearest-atom distance. (**D**) Distribution of ASA scores. The five residues with high ASA scores, fewer neighboring atoms, and shorter nearest-atom distances are highlighted in (**B**) and (**C**). Hydrophobic residues are shown in orange, and polar residues are shown in cyan.

Residue conservation and solvent-accessible surface area (ASA) were analyzed based on the predicted three-dimensional structure of OR45a (Supporting Information Figure S3). 28 residues were identified as highly conserved (conservation ≥ 0.9) and were distributed across extracellular loops, together with the extracellular ends of TM3–TM7 regions (Figure 5 and Figure 6).

ASA analysis revealed substantial variation in surface accessibility among the conserved residues, with ASA values ranging from 66.5 to 100.0. Ile146 displayed the highest ASA score (100.0) and the lowest number of neighboring Cα atoms (6; Figure 6), indicating that it is the most solvent-exposed residue. Leu122 (ASA 90.1, 8 neighbors), Tyr107 (ASA 90.1, 8 neighbors), Thr103 (ASA 90.0, 8 neighbors), and Val114 (ASA 90.0, 8 neighbors) also exhibited high solvent accessibility. In contrast, other conserved residues displayed lower ASA values (Figure 6). Across all analyzed residues, the average number of neighboring atoms was 10.4 and the mean minimum distance to the nearest atom was 3.8 Å.

### 3.6. Results of Site-Directed Mutagenesis and Functional Validation

**Figure 7:**
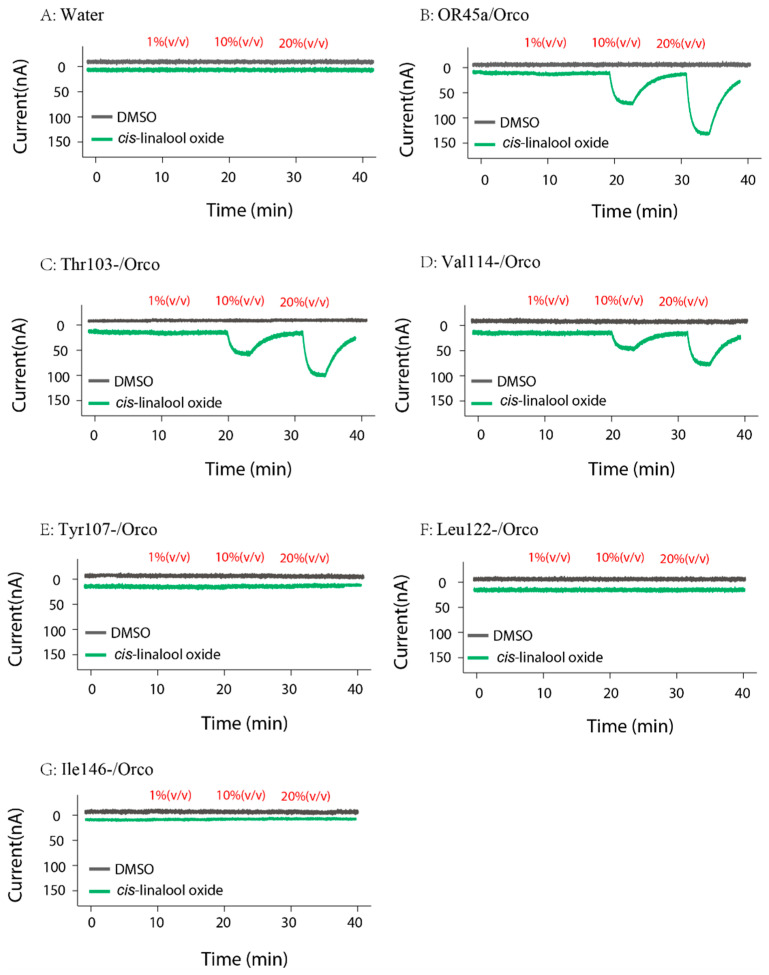
TEVC recordings of water-treated (**A**), wild-type (**B**), and five site-directed mutants (**C**–**G**) under three concentrations of cis-linalool oxide. DMSO (dimethyl sulfoxide) was used as the solvent control.

**Figure 8:**
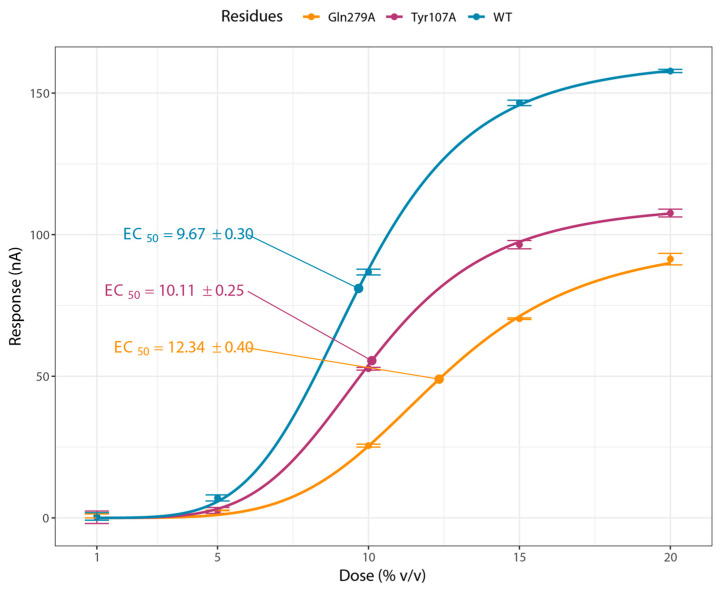
Dose–response curves of wild-type OR45a/Orco and two mutant variants.

Western blot analysis (Figure S5A) and the corresponding band intensity ratios of each mutant protein (Figure S5B) showed that the relative abundances of different mutants differed significantly (one-way ANOVA, F = 44.49, df = 7, *p* < 0.001). The wild-type OR45a–ORco complex exhibited the highest level of expression, whereas Tyr107–ORco displayed significantly lower protein levels, comparable to those of the other mutant complexes. Both the Western blot and the quantitative intensity analysis indicated that Ile146, Leu122, Val114, and Thr103 were successfully expressed, although their relative levels were somewhat lower than that of the water-treated OR45a–ORco control. In contrast, Tyr107 showed no detectable expression. The functional roles of conserved residues were examined by TEVC recordings in *X*. *laevis* oocytes expressing wild-type OR45a/Orco or point mutants (Thr103, Tyr107, Val114, Leu122, Ile146). No currents were detected in water-injected oocytes (Figure 7A). Stimulation with cis-linalool oxide (1, 10, 20% *v*/*v*) evoked robust, concentration-dependent inward currents in the wild type at 10% and 20% (Figure 7B). Mutants Thr103 (Figure 7C) and Val114 (Figure 7D) retained partial responses but showed reduced maximal currents compared with the wild type. In contrast, Tyr107, Leu122, and Ile146 failed to elicit significant responses compared with the DMSO control (Figure 7E–G). Dose–response analysis yielded Imax values of 142 ± 19 nA for the wild type, 111 ± 9 nA for Thr103, and 98 ± 10 nA for Val114. EC_50_ estimates were 9.67 ± 0.30 μL/mL for the wild type, 12.34 ± 0.40 μL/mL for Thr103, and 10.11 ± 0.25 μL/mL for Val114 (Figure 8).

## 4. Discussion and Conclusions

This study presents a molecular and functional characterization of the OR45a receptor in female *B. dorsalis*, suggesting its role in mediating attraction to the female-specific volatile, cis-linalool oxide, a chemical previously identified from *P. lilacinus* honeydew and demonstrated to effectively attract female flies [24]. Spatiotemporal expression analysis revealed that *OR45a* expression was markedly upregulated in female antennae, peaking when exposed to cis-linalool oxide for 10 days. Functional validation through RNA interference (RNAi) confirmed that silencing *OR45a* significantly reduced its transcript abundance, which correlated with diminished female attraction to cis-linalool oxide. Site-directed mutagenesis combined with two-electrode voltage-clamp (TEVC) recordings further identified Tyr107, Leu122, and Ile146 as essential residues for ligand recognition. Together, these findings support the hypothesis that *OR45a* contributes to female chemotaxis toward cis-linalool oxide.

The female-biased expression of *OR45a* likely reflects an evolutionary adaptation related to reproductive success in *B. dorsalis*. The temporal expression pattern of *OR45a* reached its peak after 10 days of exposure to cis-linalool oxide, coinciding with the oviposition period in this species. In tropical and subtropical regions, *B. dorsalis* typically begins mating and oviposition 5–7 days after eclosion, extending from 7 to 15 days depending on temperature and population density. Such synchronization suggests that *OR45a* upregulation is functionally linked to females’ need to locate suitable oviposition sites. cis-Linalool oxide, a volatile compound found in many ripening fruits [27,28], may signal fruit maturity and suitability for egg deposition. Although no definitive studies have confirmed its emission from preferred host plants of *B. dorsalis*, the evolution of female-specific receptors for such compounds would confer selective advantages by allowing gravid females to discriminate optimal oviposition substrates, thereby enhancing offspring survival [29,30].

Interestingly, *OR45a* expression in *B. dorsalis* was nearly undetectable in both the treatment and control groups after 1 day of exposure across all tissues. After 5, 10, and 15 days of exposure, however, low expression levels appeared in the control group, particularly in the antennae, although these levels remained significantly lower than those in the cis-linalool oxide–exposed *B. dorsalis*. This pattern suggests that *B. dorsalis* maintains basal expression of *OR45a* even without external stimulation, potentially preserving minimal sensitivity to cis-linalool oxide or other host-related volatiles.

Previous studies have shown that insects often retain basal expression of odorant receptors (ORs) and odorant-binding proteins (OBPs) to sustain sensitivity to environmental cues. Upon odorant exposure, many species exhibit inducible upregulation of OR and OBP genes in a time- and concentration-dependent manner, as observed in *Aedes aegypti* exposed to 1-octen-3-ol and *Holotrichia oblita* exposed to plant attractants [31,32]. These findings support a feedback mechanism whereby odorant detection modulates receptor expression, allowing dynamic tuning of olfactory sensitivity.

Our results therefore suggest that *OR45a* expression is developmentally regulated but can also be further induced by odorant exposure, indicating a combination of constitutive and inducible regulation mechanisms. Ecologically, such inducible upregulation may allow insects to adjust their olfactory systems to fluctuating environments [33]. For female *B. dorsalis*, enhanced *OR45a* expression following exposure likely enhances host-odor detection during oviposition or mate-searching. Collectively, this represents a biologically meaningful case of olfactory plasticity rather than an experimental artifact.

Comparative studies in other insects reinforce the notion that OR45a may function as a receptor for plant-derived volatiles. In the silkworm (*Bombyx mori* Linnaeus), OR45a belongs to a group of female-biased receptors with higher antennal transcript levels in females than in males [34]. Functional assays demonstrated that BmOR45 responds to several plant volatiles, including benzoic acid, 2-phenylethanol, and benzaldehyde, whereas its co-expressed partner BmOR19 is tuned to linalool. Although cis-linalool oxide has not been directly tested in *B. mori*, the responsiveness of OR45a to plant-derived volatiles and the known behavioral relevance of linalool and its oxides suggest that OR45a upregulation may be linked to female perception of host volatiles and oviposition site selection [34].

It is important to note that insect odorant receptor (OR) nomenclature is species-specific; receptors sharing the same number (e.g., “OR45a”) across species are not necessarily orthologous and may differ in evolutionary origin and function [35,36]. Similar ligand tuning—where different ORs detect related odorants—can evolve independently, a phenomenon known as convergent evolution [36,37]. Indeed, in beetles and moths, distinct ORs from separate gene lineages can detect identical or similar compounds despite lacking direct homology [37].

Consistent with this pattern, several ORs in other species respond to cis-linalool oxide or linalool. In *Apolygus lucorum*, AlucOR47 is highly sensitive to linalool [38], whereas in *Anopheles gambiae*, OR29 detects both linalool and cis-linalool oxide [39]. These findings suggest that *B. dorsalis* OR45a may also recognize linalool or structurally related derivatives. Nevertheless, this hypothesis warrants further behavioral and receptor–ligand assays to clarify evolutionary diversification in odorant receptor function. Given the structural distinctions between linalool (an acyclic monoterpene alcohol) and linalool oxides (cyclic epoxides) [40], their binding affinities to OR45a may differ substantially. Future analyses testing OR45a against both linalool enantiomers ((R) − (−) and (S) − (+)) and all four linalool oxide stereoisomers (furan/pyran, cis/trans) will help determine its ligand sensitivity and potential cross-reactivity.

From an evolutionary standpoint, linalool-responsive ORs reported in Hemiptera (*A. lucorum*, AlucOR47) and Diptera (*A. gambiae*, AgOR29), together with the linalool oxide–responsive OR45a in *B. dorsalis*, likely represent lineage-specific receptor expansions rather than a shared orthologous group [41]. Thus, AlucOR47 would not cluster within the tephritid OR45a clade, consistent with convergent evolution of linalool detection.

To our knowledge, this is the first study demonstrating that silencing *OR45a* reduces female attraction to cis-linalool oxide. Previous research on this compound has primarily focused on its ecological role in plants, where it mediates plant–insect interactions. For example, in *Arabidopsis*, *CYP76C1* regulates linalool oxide biosynthesis, reducing floral attractiveness to insects [39]. In insects, although *OBPs* differ functionally from *ORs*, silencing specific *OBPs* such as *HparOBP3* can also reduce behavioral responses to volatiles [41]. In line with these observations, our RNAi results provide direct evidence that *OR45a* mediates female recognition of cis-linalool oxide in *B. dorsalis*.

Nonetheless, RNA interference (RNAi) can induce off-target physiological effects, such as immune activation or stress responses caused by double-stranded RNA (dsRNA), potentially resulting in increased mortality. Elevated reactive oxygen species (ROS) levels and stress gene upregulation have been reported under similar conditions [42]. Moreover, RNAi efficiency and specificity can be influenced by immune reactions or dsRNA degradation, potentially complicating interpretation [43]. Thus, appropriate controls—such as assessing flight ability—are necessary to ensure that observed phenotypes reflect target-specific silencing rather than systemic stress [44]. In this study, RNAi-treated flies showed no signs of wing deformation or impaired flight behavior in wind tunnel assays. Moreover, preliminary tests revealed that *dsOR45a* individuals showed normal attraction to 1% fresh orange flavor concentrate (Shenzhen Censin Flavor & Fragrance Co., Ltd.), which was comparable to that of normal and water-injected individuals (Figure S6). Taken together, these observations indicate that the phenotypic effects observed in RNAi-treated flies were due to the specific knockdown of *OR45a*, rather than to physiological damage, stress responses of dsRNA injection.

Phylogenetic and structural analyses further support this interpretation. OR45a is highly conserved within the Tephritidae family and most closely related to the homolog in *A. ludens*, while diverging from Drosophilid homologs—likely reflecting host-specific ecological adaptation [45]. Structural modeling indicates that OR45a comprises 356 amino acids with seven inverted transmembrane helices, an intracellular N-terminus, an extracellular C-terminus, and three extracellular loops, consistent with canonical insect OR topology [46,47,48,49]. The extracellular loops (ECLs) and termini of TM3–TM7 likely form candidate ligand-binding regions, with ECL2 acting as a “lid” over the binding pocket to regulate selectivity and sensitivity [50,51].

Our TEVC results provide mechanistic insight into the selective recognition of cis-linalool oxide by OR45a, consistent with classical principles of molecular recognition, where hydrophobic interactions establish affinity and polar interactions confer specificity [52,53]. Mutational analysis identified Tyr107, Leu122, and Ile146 as key residues. Ile146 and Leu122 likely form a hydrophobic core stabilizing the ligand’s nonpolar regions via hydrophobic and van der Waals interactions, while Tyr107 provides specificity and additional stabilization through hydrogen bonding and π interactions. Ligand binding thus likely occurs in two stages: hydrophobic anchoring followed by Tyr107-mediated locking, resulting in high affinity and selectivity. This dual-residue synergy underscores the interplay of hydrophobic and polar forces in defining receptor specificity [54,55,56].

Single-point mutants exhibited reduced sensitivity (rightward EC_50_ shift) and a diminished maximal response, whereas the triple mutant (Tyr107, Leu122 and Ile146) completely lost responsiveness, confirming the collective importance of these residues for ligand recognition. Western blot analysis revealed that the Leu122 and Ile146 mutants, along with the wild type, displayed clear bands at approximately 46 kDa, whereas the Tyr107 mutant showed no detectable band. This suggests that, among the three mutants lacking TEVC responses, the Leu122 and Ile146 substitutions likely affect ligand-binding interactions without severely disrupting receptor folding or stability. In contrast, the Tyr107 mutation may critically impair protein folding, stability, or post-translational processing, although its potential involvement in ligand recognition cannot be excluded.

The rightward EC_50_ shift indicates reduced ligand sensitivity, while the lower maximal response reflects compromised receptor activation, possibly due to altered channel gating or weakened interaction with the Orco co-receptor. These findings suggest that the substituted residues participate not only in ligand recognition but also in receptor activation dynamics [57]. Nevertheless, since heterologous expression systems may not fully recapitulate the native cellular context—such as the presence of accessory proteins or specific post-translational modifications—these quantitative differences should be interpreted with caution [58,59]. Considering that these residues are located within the predicted ligand-binding cavity rather than the transmembrane regions required for receptor assembly, their substitution most likely disrupts ligand docking specifically. Future studies using additional odorants (e.g., hexanol, ethyl phenylacetate) and in vivo calcium imaging [58] or single-sensillum recordings [59] will further validate this mechanistic model.

In summary, this study provides molecular and functional evidence that *OR45a* mediates female-specific attraction to the volatile compound *cis*-linalool oxide in *Bactrocera dorsalis*. *OR45a* was found to be highly expressed in female antennae during the reproductive period, and its silencing markedly reduced female responsiveness to *cis*-linalool oxide, suggesting that this receptor is involved in host plant or oviposition site searching. Phylogenetic, conserved-residue, and site-directed mutagenesis analyses further revealed that Leu122 and Ile146 are critical for ligand recognition and receptor activation, underscoring the dual roles of hydrophobic and polar interactions in determining ligand specificity. In contrast, Tyr107 appears to be more important for maintaining protein stability and proper folding. Together, these findings identify *OR45a* as a key molecular determinant of female olfactory behavior and provide mechanistic insight into the evolution of odorant receptor selectivity in tephritid flies.

## Data Availability

The raw data supporting the conclusions of this article will be made available by the authors on request.

## Supplementary Materials

The following supporting information can be downloaded at: https://www.mdpi.com/article/10.3390/insects16111139/s1, Table S1: species for *OR45a* phylogenetic analysis; Table S2: Primers for heterologous expression and site-directed mutagenesis; Figure S1: Schematic diagram of the wind-tunnel system used to evaluate the olfactory responses of *Bactrocera dorsalis* females to cis-linalool oxide. In the odor-source zone, a 10 cm glass Petri dish was placed centrally at the bottom of the cage. A total of 5 mL of 10% (*v*/*v*) cis-linalool oxide solution (dissolved in dimethyl sulfoxide, DMSO) was added to the dish. The dish was sealed with Parafilm M and perforated with thirty evenly spaced holes (1–2 mm in diameter) to allow controlled volatile release; Figure S2: Three-dimensional structure of *B. dorsalis* OR45a. The protein structure was predicted by AlphaFold2, with α-helices shown in red, forming the core scaffold and transmembrane domains. Loop regions are shown in gray, connecting α-helices and potentially contributing to ligand binding or gating mechanisms. Short β-sheet-like structures are displayed in green, sporadically located between helices. The transmembrane helix bundle, a tightly packed functional core unit composed of multiple α-helices, embeds within the cell membrane, forming the basis for ion channels and odorant-binding sites; Figure S3: Structural confidence and multiple sequence alignment (MSA) quality of the predicted protein (*B. dorsalis* OR45a) model by AlphaFold2. (A) Sequence coverage plot showing the depth and identity of multiple sequence alignments (MSA) used for structure prediction. Each row represents a matching sequence; the color scale indicates sequence identity to the query (0 to 1), and the black line shows the number of aligned sequences at each residue position. (B) Predicted distance/contact maps for the top five ranked models (rank_1 through rank_5). High-intensity points in the matrix indicate predicted spatial proximity between residue pairs. (C) Predicted local distance difference test (pIDDT) confidence scores per position for each ranked model. High pIDDT values (>70) suggest high model confidence, while dips in the curves correspond to structurally uncertain or disordered regions; Figure S4: Transmembrane topology prediction of *B. dorsalis* OR45a using multiple algorithms, compiled by TOPCONS. Top panel: Transmembrane topology was predicted by six computational tools (TOPCONS, OCTOPUS, Philius, PolyPhobius, SCAMPI, and SPOCTOPUS). Red and blue horizontal bars indicate regions predicted to be located inside or outside the membrane, respectively. Gray and white boxes indicate predicted transmembrane helices with IN→OUT or OUT→IN orientation, respectively, while black boxes represent signal peptides, where applicable. All tools consistently predict seven transmembrane helices with concordant topology, suggesting the presence of a canonical 7-TM structure. Middle panel: No homologous transmembrane proteins were identified in the PDB database, suggesting that this protein may adopt a novel fold or possess an uncharacterized membrane topology. Bottom panel: Predicted free energy changes (ΔG) for membrane insertion are shown along the sequence, with local minima corresponding to likely transmembrane regions; Figure S5: Western blot analysis (A) and corresponding band intensity ratios of each mutant protein (B). The relative abundances of different mutants differed significantly (one-way ANOVA, F = 44.49, *df* = 7, *p* < 0.001). The wild-type (WT) OR45a/ORco complex showed the highest level of expression, whereas Tyr107/ORco exhibited significantly lower protein levels, comparable to those of the other mutant complexes. Both the Western blot and the quantitative intensity analysis indicate that Ile146, Leu122, Val114, and Thr103 were successfully expressed, although their relative levels differed somewhat from the wild-type OR45a/ORco control, while Tyr107 showed no detectable expression, suggesting that the Tyr107 residue is critical for OR45a protein stability; Figure S6: Differences in female attraction to fresh orange flavor concentrate were compared among four treatments (Control, Water, *dsGFP*, and *dsOR45a*). The Kruskal–Wallis test showed no significant differences among groups (χ^2^ = 4.62, *df* = 3, *p* = 0.202), suggesting that the treatments had no substantial effect on the attraction index, defined as the proportion of flies choosing the odor source side (with water on the control side) out of the total tested.
