# Supplementary material for: Odorant Receptor OR45a Mediates Female-Specific Attraction to cis-Linalool Oxide in Bactrocera dorsalis
Source: Insects. 2025 Nov 7;16(11):1139. doi: 10.3390/insects16111139 (PMC12653480; doi:10.3390/insects16111139)
Supplement: Supplementary file 1 [file insects-16-01139-s001.zip › insects-3891285-supplementary.pdf]

Table S1 species for *OR45a* phylogenetic analysis

Index	Name	Length
1	*Drosophila busckii*	315
2	*Drosophila suzukii*	382
3	*Drosophila pseudoobscura*	381
4	*Drosophila bipectinata*	379
5	*Drosophila kikkawai*	415
6	*Drosophila takahashii*	382
7	*Drosophila tropicalis*	378
8	*Drosophila melanogaster*	378
9	*Anastrepha ludens*	390
10	*Lucilia cuprina*	368
11	*Bactrocera dorsalis*	365

Table S2 Primers for heterologous expression and site-directed mutagenesis

Mutation Protein Position	Wild-type Codon (CDS)	Mutant Codon	Forward Primer (5'→3') Mutant Codon in [ ]	Reverse Primer (5'→3') Complementary Reverse	Length (nt)	GC Count	Approximated Tm (Wallace)
Thr103	CTG (Leu)	GCT	GCTGAGAATATTTAT[GCT]ATATCGACCAGAGCC	GGCTCTGGTCGATATAGCATAAATATTCTCAGC	33	14	94 °C
Tyr107	AGA (Arg)	TTT	TATCTGATATCGAC[TTT]GCCTCTGCGAAGGAG	CTCCTTCGCAGAGGCAAAGGTCGATATCAGATA	33	16	98 °C
Val114	ACT (Thr)	GCT	TCTGCGAAGGAG[GCT]CACTTGATACAGGAG	CTCCTGTATCAAGTGAG[AGC]TAGCTCCTTCGCAGA	33	17	100 °C
Leu122	CGT (Arg)	GCT	ATACAGGAGAATAAT[GCT]GAGCGTCTAATGAAC	GTTCATTAGACGCTCAGCATTATTCTCCTGTAT	33	13	92 °C
Ile146	GTT (Val)	GCT	GCGCTTGCGGCCCCC[GCT]TTGGTGAGCTTCATA	TATGAAGCTCACCAAAGCGGGGGCCGCAAGCGC	33	21	108 °C
OR45a (Cloning)	-	-	CGCGGATCCGCCACCATGTTCAAGAGCGATTTGGG	CCGCTCGAGTCATTCGCACGCATATTCAAG	F:35/R:30	F:21/R:16	92°C
ORco (Cloning)	-	-	CCGGAATTCCGCCACCATGGTTGGTGTTTGATTGAT	ATAAGAATGCGGCCGCTTACGCGTAGGCAGTTGGTA	F:36/R:36	F:18/R:19	108°C

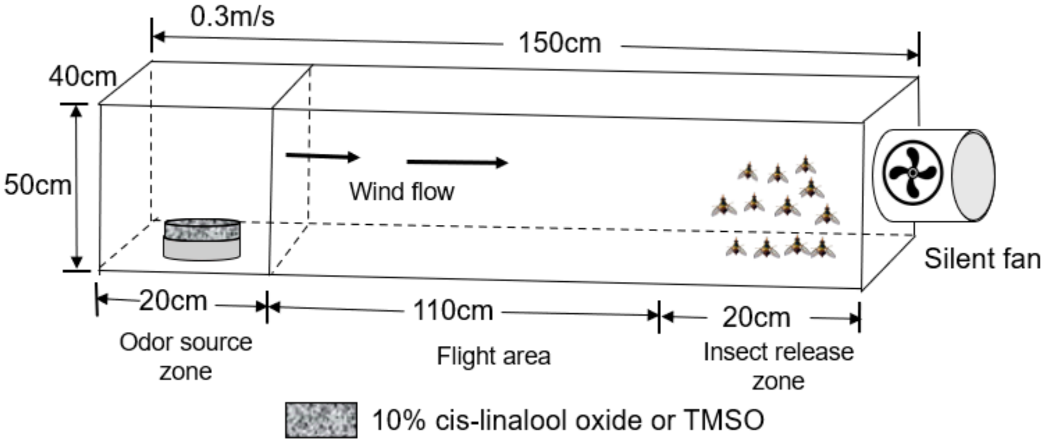

Figure S1 Schematic diagram of the wind-tunnel system used to evaluate the olfactory responses of *Bactrocera dorsalis* females to cis-linalool oxide. In the odor-source zone, a 10 cm glass Petri dish was placed centrally at the bottom of the cage. A total of 5 mL of 10% (v/v) cis-linalool oxide solution (dissolved in dimethyl sulfoxide, DMSO) was added to the dish. The dish was sealed with Parafilm M and perforated with thirty evenly spaced holes (1–2 mm in diameter) to allow controlled volatile release.

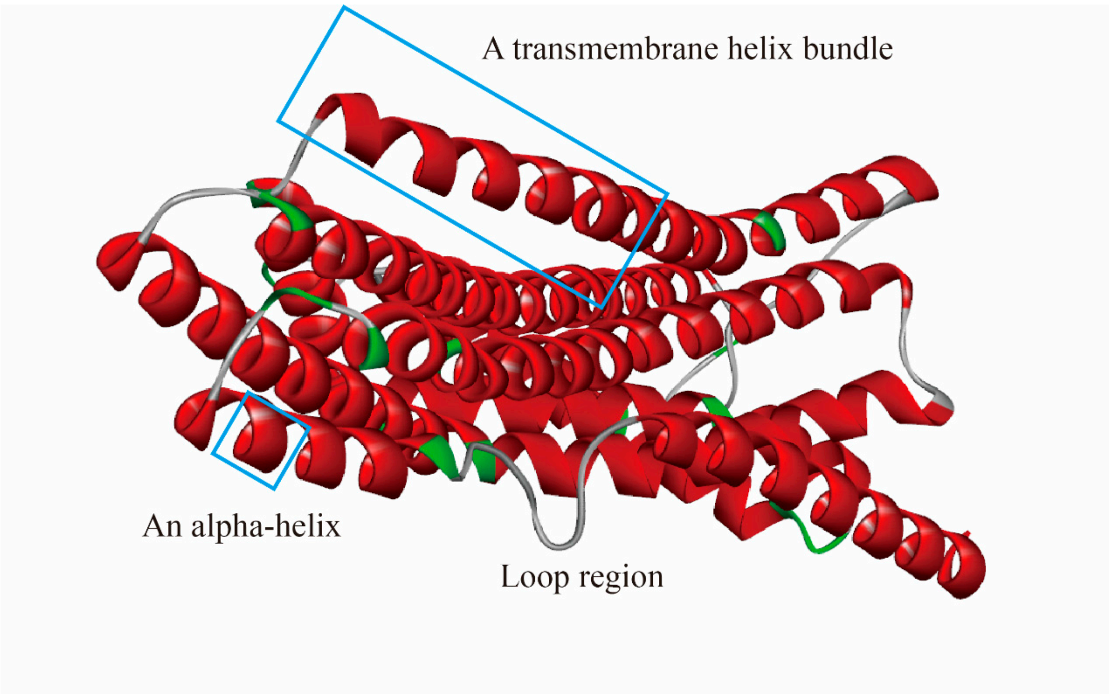

Figure S2 Three-dimensional structure of *B. dorsalis* OR45a. The protein structure was predicted by AlphaFold2, with α-helices shown in red, forming the core scaffold and transmembrane domains. Loop regions are shown in gray, connecting α-helices and potentially contributing to ligand binding or gating mechanisms. Short β-sheet-like structures are displayed in green, sporadically located between helices. The transmembrane helix bundle, a tightly packed functional core unit composed of multiple α-helices, embeds within the cell membrane, forming the basis for ion channels and odorant-binding sites.

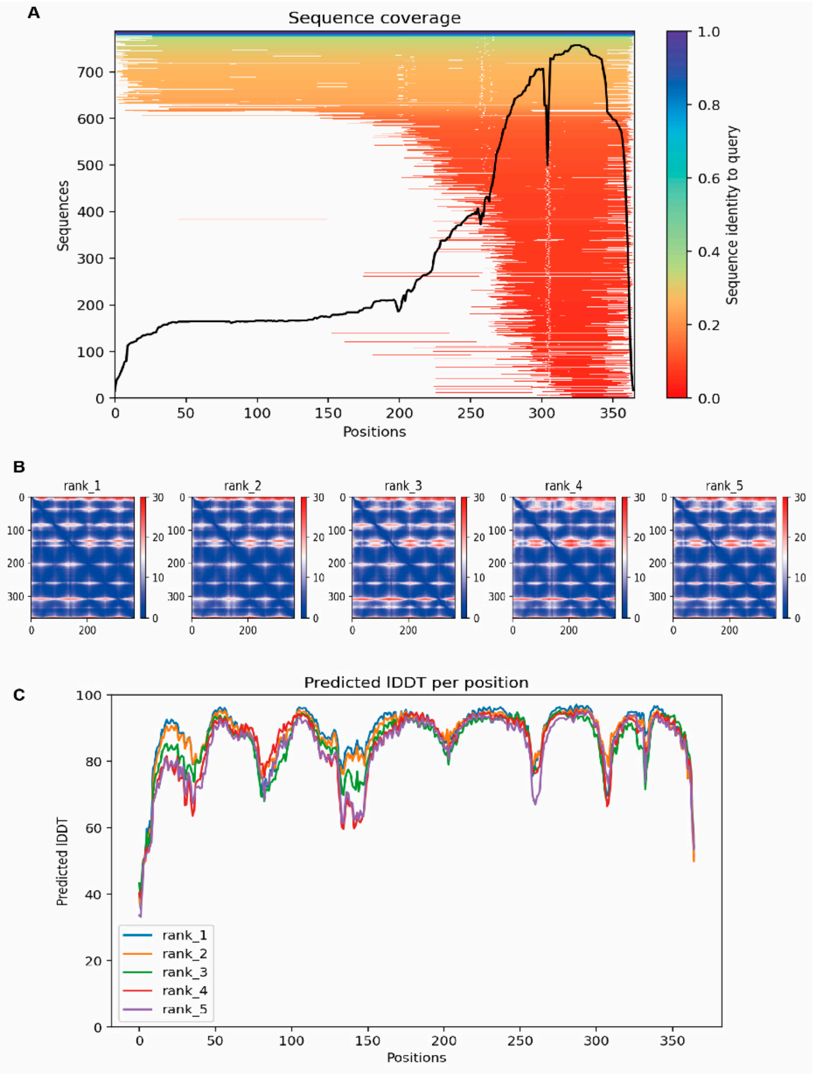

Figure S3 Structural confidence and multiple sequence alignment (MSA) quality of the predicted protein (*B. dorsalis* OR45a) model by AlphaFold2. (A) Sequence coverage plot showing the depth and identity of multiple sequence alignments (MSA) used for structure prediction. Each row represents a matching sequence; the color scale indicates sequence identity to the query (0 to 1), and the black line shows the number of aligned sequences at each residue position. (B) Predicted distance/contact maps for the top five ranked models (rank_1 through rank_5). High-intensity points in the matrix indicate predicted spatial proximity between residue pairs. (C) Predicted local distance

difference test (pIDDT) confidence scores per position for each ranked model. High pIDDT values (>70) suggest high model confidence, while dips in the curves correspond to structurally uncertain or disordered regions.

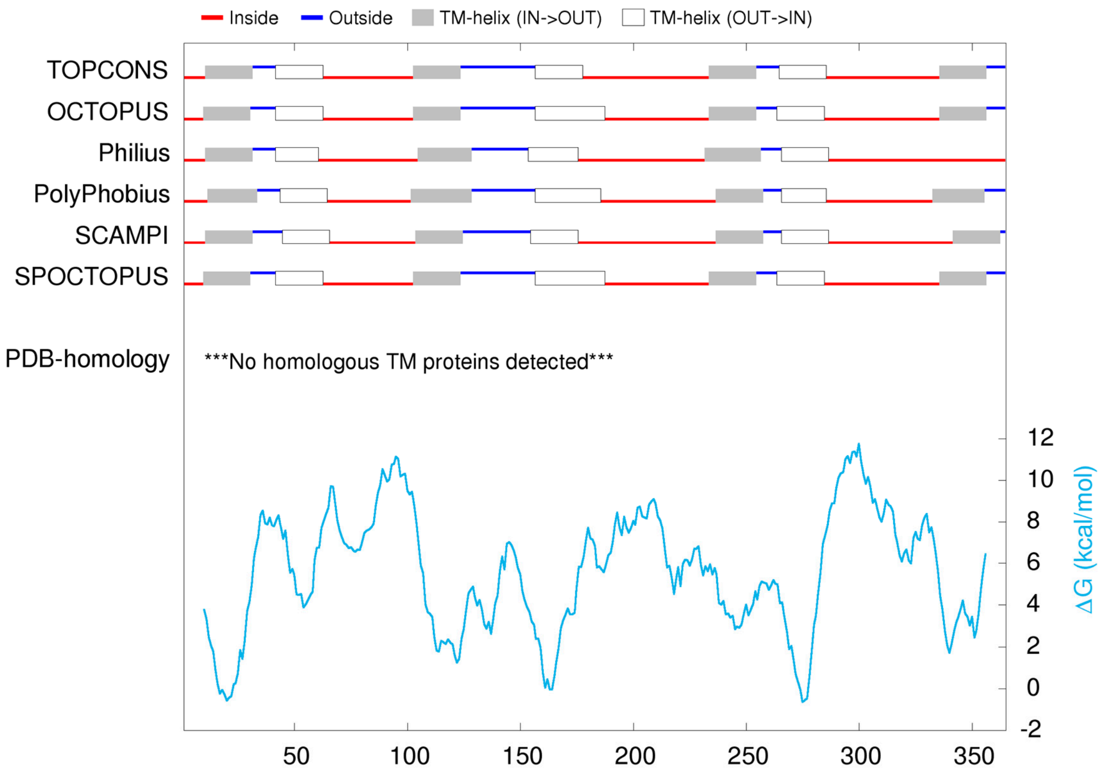

Figure S4 Transmembrane topology prediction of *B. dorsalis* OR45a using multiple algorithms, compiled by TOPCONS. Top panel: Transmembrane topology was predicted by six computational tools (TOPCONS, OCTOPUS, Philius, PolyPhobius, SCAMPI, and SPOCTOPUS). Red and blue horizontal bars indicate regions predicted to be located inside or outside the membrane, respectively. Gray and white boxes indicate predicted transmembrane helices with IN→OUT or OUT→IN orientation, respectively, while black boxes represent signal peptides, where applicable. All tools consistently predict seven transmembrane helices with concordant topology, suggesting the presence of a canonical 7-TM structure. Middle panel: No homologous transmembrane proteins were identified in the PDB database, suggesting that this protein may adopt a novel fold or possess an uncharacterized membrane topology. Bottom panel: Predicted free energy changes (ΔG) for membrane insertion are shown along the sequence, with local minima corresponding to likely transmembrane regions.

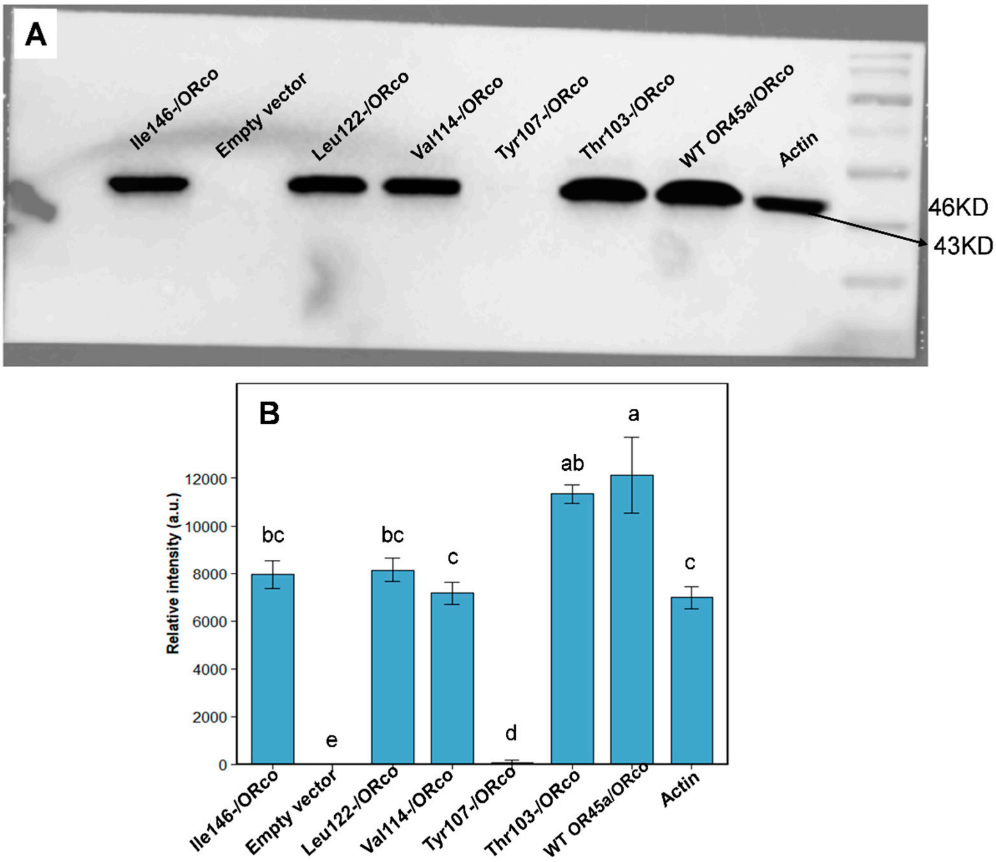

Figure S5 Western blot analysis (A) and corresponding band intensity ratios of each mutant protein (B). The relative abundances of different mutants differed significantly (one-way ANOVA, $F = 44.49$, $df = 7$, $p < 0.001$). The wild-type (WT) OR45a/ORco complex showed the highest level of expression, whereas Tyr107/ORco exhibited significantly lower protein levels, comparable to those of the other mutant complexes. Both the Western blot and the quantitative intensity analysis indicate that Ile146, Leu122, Val114, and Thr103 were successfully expressed, although their relative levels differed somewhat from the wild-type OR45a/ORco control, while Tyr107 showed no detectable expression, suggesting that the Tyr107 residue is critical for OR45a protein stability. Bars with different lowercase letters differ significantly among treatments (Tukey's HSD test, $p < 0.05$).

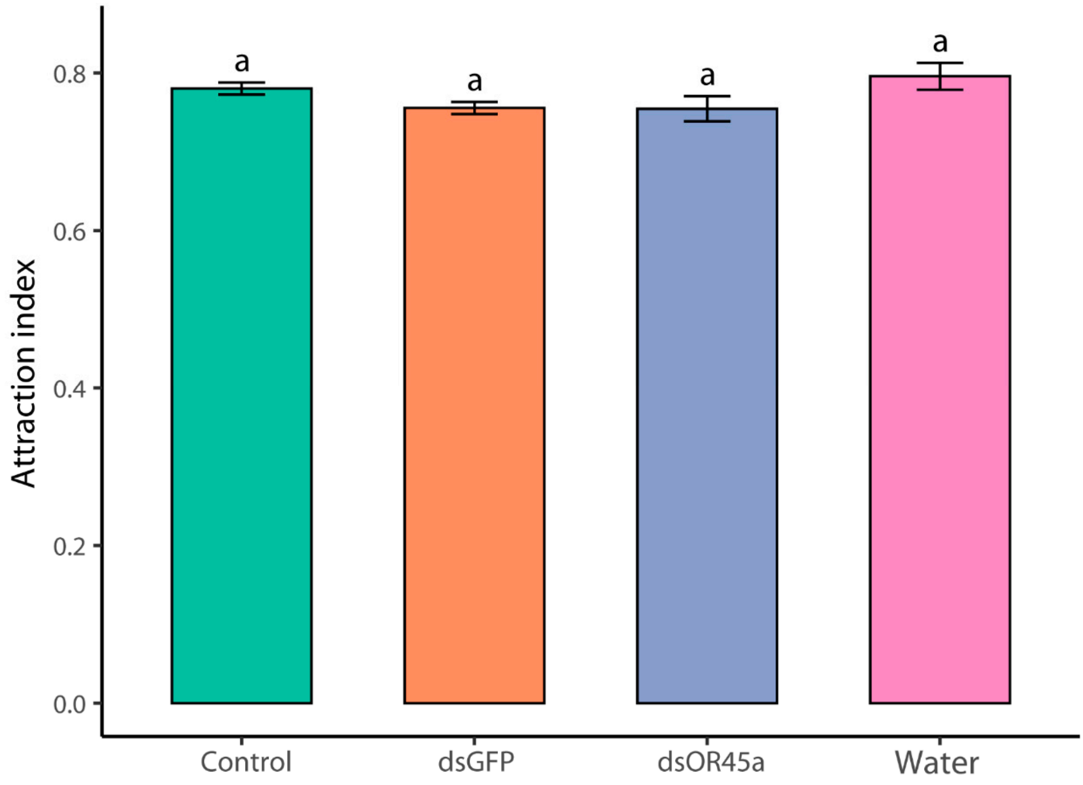

Figure S6 Differences in female attraction to fresh orange flavor concentrate were compared among four treatments (Control, Water, *dsGFP*, and *dsOR45a*). The Kruskal–Wallis test showed no significant differences among groups ($\chi^2 = 4.62$, $df = 3$, $p = 0.202$), suggesting that the treatments had no substantial effect on the attraction index, defined as the proportion of flies choosing the odor source side (with water on the control side) out of the total tested. Bars with different lowercase letters differ significantly among treatments (Tukey's test, $p < 0.05$).
